# Increased Expression of Autophagy-Related Genes in Alzheimer’s Disease—Type 2 Diabetes Mellitus Comorbidity Models in Cells

**DOI:** 10.3390/ijerph20054540

**Published:** 2023-03-03

**Authors:** Clara Vianello, Marco Salluzzo, Daniela Anni, Diana Boriero, Mario Buffelli, Lucia Carboni

**Affiliations:** 1 Department of Medical and Surgical Sciences, Alma Mater Studiorum University of Bologna, Via Irnerio 48, 40126 Bologna, Italy; 2 Department of Pharmacy and Biotechnology, Alma Mater Studiorum University of Bologna, Via Irnerio 48, 40126 Bologna, Italy; 3 Department of Neurosciences, Biomedicine and Movement Sciences, University of Verona, Strada Le Grazie, 8, 37134 Verona, Italy

**Keywords:** Alzheimer’s disease, type 2 diabetes mellitus, autophagy, *ATG16L1*, *ATG16L2*, *GABARAP*, *GABARAPL1*, *GABARAPL2*, SQSTM1, neuronal cultures

## Abstract

The association between Alzheimer’s disease (AD) and type 2 diabetes mellitus (T2DM) has been extensively demonstrated, but despite this, the pathophysiological mechanisms underlying it are still unknown. In previous work, we discovered a central role for the autophagy pathway in the common alterations observed between AD and T2DM. In this study, we further investigate the role of genes belonging to this pathway, measuring their mRNA expression and protein levels in 3xTg-AD transgenic mice, an animal model of AD. Moreover, primary mouse cortical neurons derived from this model and the human H4Swe cell line were used as cellular models of insulin resistance in AD brains. Hippocampal mRNA expression showed significantly different levels for *Atg16L1*, *Atg16L2*, *GabarapL1*, *GabarapL2*, and *Sqstm1* genes at different ages of 3xTg-AD mice. Significantly elevated expression of *Atg16L1*, *Atg16L2*, and *GabarapL1* was also observed in H4Swe cell cultures, in the presence of insulin resistance. Gene expression analysis confirmed that *Atg16L1* was significantly increased in cultures from transgenic mice when insulin resistance was induced. Taken together, these results emphasise the association of the autophagy pathway in AD-T2DM co-morbidity, providing new evidence about the pathophysiology of both diseases and their mutual interaction.

## 1. Introduction

Alzheimer’s disease (AD) is the most common cause for dementia, and 55 million people are estimated to live with this condition worldwide. Numbers are expected to increase to 113 million by 2050 [1], causing enormous impacts on global health and imposing a huge economic burden. Therapeutic approaches encompass cholinesterase inhibitors and memantine as symptomatic agents [2,3]. Great hopes have been raised by antibodies which target amyloid-beta aggregates in the brain as potential disease-modifying interventions [4,5]. However, whether meaningful clinical efficacy can be reached as well as cost-effectiveness are still questions, while safety concerns need further analyses and clarification [6,7,8]. Therefore, preventive actions directed at potentially modifiable risk factors are crucial to reduce AD severe disease burden [2].

Both genetic and environmental factors contribute to AD risk. Dominantly inherited mutations in *APP*, *PSEN1*, and *PSEN2* genes account for rarer early-onset cases, whereas carrying at least one copy of the *APOE* ε4 allele is the strongest genetic risk factor for the common late-onset form [9]. Although age is the most relevant factor providing the largest impact, additional environmental components present important contributions that are potentially modifiable [2]. Among the latter, consistent evidence supports major roles for education, hypertension, obesity, hearing loss, traumatic brain injury, smoking, depression, physical inactivity, social isolation, type 2 diabetes mellitus (T2DM), and air pollution as potential targets of intervention in different life stages, particularly at midlife [2]. In addition, T2DM has been compellingly associated with significantly greater risk of dementia [10,11,12]. Moreover, metabolic syndrome and obesity, which are often associated with T2DM, represent dementia risk factors per se, thus further complicating the picture [13,14]. It has been suggested that since T2DM is modifiable, its reduction could constitute a possible strategy for reducing future AD incidence. Indeed, it has been estimated that if T2DM was removed as a risk factor, about 1.1% of dementia cases could be prevented. Although this percentage is low, the number of impacted people is nonetheless high when considering global incidence rates [12].

Despite the demonstrated convincing association between AD and T2D, the pathophysiological mechanisms responsible are still unknown. As a result, the best approach to be adopted for prevention still needs to be elucidated [12]. Furthermore, whether antidiabetic treatments represent a useful way forward is uncertain at present, as available data are inconsistent [2,15,16]. Several hypotheses have been proposed to explain the mechanistic link between AD and T2DM [17,18,19]. Among them, insulin signalling is impaired in both AD and T2DM, and the definition of AD as type 3 diabetes is based on the observed insulin resistance [20,21,22,23]. In addition, defects in mitochondrial function are shared by both AD and T2DM, thus a common causative role has been proposed for this defect based on preclinical and clinical findings [24,25]. In a previous study, we adopted a system biology approach to address this important gap in knowledge about the common pathophysiological dysregulations contributing to AD and T2DM comorbidity. We compared molecular mechanistic networks underlying brain T2DM pathophysiology in AD and control subjects by analysing transcriptional datasets with a novel approach. We discovered a central role for the autophagy pathway in the mechanisms shared between AD and T2DM [26]. Autophagy is an intracellular degradation pathway that traffics organelles, dysfunctional proteins, and infectious agents to lysosomes via specific vesicles called autophagosomes [27]. In agreement with our findings, autophagy relevance in AD is supported by a wealth of data, and targeting this mechanism is proposed as a potential avenue for drug discovery [28,29,30]. Moreover, abnormal autophagic responses have been implicated in metabolic disorders [31].

The aim of this study was to further investigate the role of genes identified in our previous studies as relevant for the pathophysiology of Alzheimer’s disease and T2DM comorbidity, namely *ATG16L1*, *ATG16L2*, *GABARAP*, *GABARAPL1*, *GABARAPL2*, and *SQSTM1*. We thus investigated the modulation of these genes in an animal model of AD and in cellular models of insulin resistance in Alzheimer’s disease brains.

## 2. Materials and Methods

### 2.1. Antibodies

In immunofluorescence experiments, the following antibodies and dilutions were used: anti-Phospho SQSTM1/p62 (S349) (Abcam, Cambridge, UK, cat # ab211324) 1:100; anti-SQSTM1/p62 (Abcam, cat # ab56416) 1:50; anti-β-Tubulin III (Merck Millipore, Burlington, MA, USA cat # T2200) 1:500; anti-MAP2 (1:500, Merck Millipore, cat # M9942) 1:500; anti-GFAP (Thermo Fisher Scientific, Waltham, MA, USA, cat # 13-0300), 1:800, donkey anti-rabbit-IgG Alexa Fluor 488 (Thermo Fisher Scientific, cat # R37118) 1:1000; donkey anti-mouse-IgG Alexa Fluor 594 (Thermo Fisher Scientific, cat # A-21203, 1:1000); goat anti-mouse IgG1 CF 568 (Merck, cat # SAB4600313 1:1000); goat anti-rat Alexa Fluor 647 (Thermo Fisher Scientific, cat # A21247, 1:1000); and DAPI (4′,6-diamidino-2-phenylindole Merck Millipore, cat #D9542) 1:5000. In Western blotting experiments, the following antibodies and dilutions were used: anti-Phospho SQSTM1/p62 (S349) (Abcam cat # ab211324) 1:2000; anti-SQSTM1/p62 (Abcam cat # ab56416) 1:2000; Anti-GAPDH (Abcam cat # ab8245); 1:5000; anti-phospho-Akt (Ser473 D9E Cell Signaling, Danvers, MA, USA, cat #4060) 1:2000; anti-Akt (Cell Signaling, cat # 9272, 1:1000); goat anti-mouse IgG IRDye 800(Li-Cor, Lincoln, NE, USA, cat # 926-32210) 1:5000; and goat anti-rabbit-IgG Alexa 680 (Thermo Fisher Scientific cat # A21076) 1:5000.

### 2.2. Animals

A colony of triple-transgenic AD mice (3xTg-AD) expressing three mutant human transgenes—PS1M146V, APPSwe, and tauP301L—was established at the University of Verona by purchasing transgenic mice from The Jackson Laboratory (Sacramento, CA, USA). C57BL/6J mice were purchased from Charles River Italia (Calco, Italia). Mice were housed at 3/cage at a constant room temperature of 21 ± 1 °C and maintained on a 12:12h light/dark cycle with lights on at 7.30 a.m. with freely available food and water. All efforts to minimise animal suffering and number were made. This study is compliant with ARRIVE guidelines [32]. Procedures involving animals were conducted in conformity with the EU guidelines (2010/63/UE) and Italian law (decree 26/14) and were approved by the University of Verona’s ethical committee and the local authority’s veterinary service. The Italian Health Ministry Ethical Committee for Protection of Animals approved the study (approval number: 283/2019-PR). For gene expression studies, 18 (six/group aged 6, 12, and 18 months) female transgenic mice and 18 (six/group) female wild-type mice were used. For immunofluorescence on brain sections, 12-month-old female 3xTg-AD and wild-type mice were used (n = 3/group). Mice for gene or protein expression experiments were anesthetised using Tribromoethanol (Merck Millipore) and sacrificed. Brain dissections were performed in Petri dishes on ice; the hippocampi were collected, flash-frozen in liquid nitrogen, and stored at −80 °C until analysis. The whole procedure did not exceed 5 min to preserve brain integrity. Mice for immunofluorescence experiments were anesthetised using Tribromoethanol, perfused transcardially with 0.1 M phosphate buffered saline solution (PBS), followed by formaldehyde 10% V/V, buffered 4% *w/v* (Titolchimica, Rovigo, Italy), and brains were extracted and postfixed overnight. Seven dams were used (wild-type: n = 4; 3xTg-AD n = 3) and neuronal cultures were prepared from 5–6 pups/preparation for each genotype.

### 2.3. Neuronal Cultures

Human glioblastoma H4 cell lines stably expressing the βAPP-Swe mutation (K595N/M596L) were a kind gift from prof. M. Pizzi, University of Brescia, Italy. Cells were cultured in DMEM with 10% foetal bovine serum (FBS), 100 Units/mL penicillin, 2 mM glutamine, and 100 μg/mL streptomycin (Thermo Fisher Scientific) [33]. After reaching 80% confluence and twenty-four hours before starting the experiment, cells were trypsinised and seeded at a density of 4 × 10^6^ cells in T75 cm^2^ flasks. Treatments were performed in DMEM medium without FBS.

### 2.4. Primary Cortical Neurons

Primary mouse cortical cultures were prepared as previously described [34] with modifications [35]. Briefly, newborn C57BL/6 and 3xTg-AD mice (P0-P1) brains were isolated and cortices were dissected in 1X ice-cold DBPS medium (cat # 14200075, Thermo Fisher Scientific). After removal of meninges, cortices were washed twice and enzymatically digested with DPBS solution containing 0.25% (*v*/*v*) trypsin (Thermo Fisher Scientific), 1 mM sodium pyruvate, 0.1% (*w*/*v*) glucose, and 10 mM HEPES pH 7.3 for 20 min at 37 °C. Following a 5 min incubation with 0.1mg/mL DNAse I (Merck Millipore) at room temperature, the enzymatic reaction was stopped with an MEM solution containing 10% FBS, 0.45% (*w*/*v*) glucose, 1 mM sodium pyruvate, 2 mM L-glutamine, 100 U/mL penicillin, and 100 μg/mL streptomycin (all reagents from Thermo Fisher Scientific). Next, the tissue was triturated through a P1000 pipette, and the cell suspension was passed through a 70 µm MACS SmartStrainer (Miltenyi Biotec, Bergisch Gladbach, Germany). Cells were then counted and diluted to 8 × 10^5^ cells/mL in Neurobasal™-A Medium (NBA, Thermo Fisher Scientific) containing 1X B27 supplement (Thermo Fisher Scientific), 2 mM L-Glutamine (Thermo Fisher Scientific), 100 U/mL penicillin, and 100 μg/mL streptomycin (Thermo Fisher Scientific) and plated on 6-well plates pre-coated with 0.1 mg/mL poly-L lysine (Merck Millipore). Cells were maintained in a standard, humidified 5% CO_2_ incubator until the day of the experiment (5–7 days in vitro, DIV).

### 2.5. Insulin Resistance

To monitor insulin response, cells were challenged with 100 nM insulin (Merck Millipore) for 30 min. To induce insulin resistance, cells were treated for 24 h with 40 mM glucose (Merck) or 20 nM insulin before receiving insulin challenge [36]. Controls were treated with vehicle. At the end of the experiments, both H4Swe cells and primary mouse neurons were washed with PBS and harvested by 5 min centrifugation at 2900× *g*, and the pellets were re-suspended in RNA later (Thermo Fisher Scientific), stored at 4 °C for 24 h, and transferred at −20 °C until RNA extraction. Treatments were repeated in 3–6 independent experiments.

### 2.6. Quantitative Real-Time PCR

Gene expression was assessed by qPCR as previously reported [37] with slight changes. RNA was extracted with the Aurum total RNA mini kit (Bio-Rad, Hercules, CA, USA) which includes a DNase I digestion step, following manufacturer’s instructions. RNA amount was assessed by UV absorbance in a NanoDrop 2000c spectrophotometer (Thermo Fisher Scientific). cDNA was synthesised using the iScript Advanced cDNA synthesis Kit (Bio-Rad). qPCR was performed by Sybr Green technology in a 7900HT Fast Real-Time PCR System (Thermo Fisher Scientific) with SSO Advanced Universal SYBR Green Supermix (Bio-Rad) in 20 µL according to this protocol: stage 1: 95 °C, 20 s; stage 2: 40 × (95 °C, 3 s; 60 °C, 30 s). Primers were selected with the NCBI Primer-BLAST tool and purchased from Eurofins Italia (Torino, Italy). Sequences are reported in Table 1. Data were analysed using the Delta-Delta-Ct method, converting to a relative ratio (2^−DDCt^) for statistical analysis [38] by normalising to the geometric average of two endogenous reference genes: *Gapdh* and *Ywhaz*, as previously reported [39,40]. The specificity of amplification products was evaluated by building a dissociation curve in the 60–95 °C range.

### 2.7. Western Blot

Hippocampi were homogenised with a micro-pestle in ice-cold lysis buffer (10% *w*/*v*) containing 50 mM Tris-HCl (pH 7.5), 2% Igepal, 10 mM MgCl_2_, 0.5 M NaCl, 2 mM EDTA, 2 mM EGTA, 5 mM benzamidine, 0.5 mM phenyl-methylsulfonyl fluoride, 8 mg/mL pepstatin A, 20 mg/mL leupeptin, 50 mM β-glycerolphosphate, 100 mM sodium fluoride, 1 mM sodium vanadate, 20 mM sodium pyrophosphate, and 100 nM okadaic acid. Homogenates were clarified by 1 min centrifugation at 10,000× *g* at 4 °C and protein concentration was assessed by Precision Red Protein Quantification Assay (Cytoskeleton). H4Swe cells were seeded into 6-well plates at a density of 9.5 × 10^5^ cells/well. Following treatments, cells were washed once in Tris-buffered saline (TBS), lysed, and assayed for protein with the Bradford method (Merck). In both instances, lysates were processed for Western blot as previously reported [41], with slight changes. Briefly, lysates were separated using 4–12% Bis-Tris gels (Novex pre-cast gel, Thermo Fisher Scientific) and transferred to 0.45 μm nitrocellulose membranes (Thermo Fisher Scientific). Blots were blocked for 1 h at room temperature in 1X Odyssey blocking buffer (TBS) and incubated with primary antibodies overnight in Odyssey blocking buffer (TBS) plus 0.1% Tween-20 (Tween-20 TBS) at 4 °C. Membranes were washed 3 × 10 min in Tween-20 TBS at room temperature, followed by incubation with secondary antibody conjugated to IRDye diluted in Tween-20 TBS for 1 h at room temperature. Blots were washed 2 × 10 min in TBST, 1 × 10 min in TBS, and visualised with Odyssey Infrared Imaging System (Li-Cor) by quantifying fluorescent signals as Integrated Intensities (I.I. K Counts) using the Odyssey Infrared Imaging System. After background subtraction, protein levels were assessed as total protein to Gapdh loading control ratios or as phosphorylated to total protein ratios.

### 2.8. Immunofluorescence

In brain sections, immunofluorescence was carried out as previously reported [42]. Briefly, after post-fixing, brains were embedded in an OCT cryoembedding matrix and sectioned on the coronal plane at 30 mm thickness with a cryostat. Sections were treated with a blocking solution of 2% bovine serum albumin, 2% normal goat serum, and 0.2% Triton X100 in PBS for 20 min at room temperature and incubated overnight at 4 °C in primary antibodies. Secondary antibodies were diluted 1:1000 in the above blocking solution, with the appropriate serum. After immunohistochemical processing, sections were counterstained with the fluorescent nuclear marker DAPI (100 ng/mL) for 10 min at room temperature and mounted on slides with 0.1% paraphenylenediamine in glycerol-based medium (90% glycerol 10% PBS). For H4Swe cell immunostaining, 5 × 10^5^ cells/well were seeded onto 18 mm round coverslips in 24-well plates and left to attach overnight. The next day, cells were washed twice with PBS and fixed with 4% paraformaldehyde for 20 min. Fixed cells were treated for 10 min with blocking and incubated overnight with primary antibodies in blocking solution. After three washes with PBS, samples were incubated with secondary antibodies diluted 1:2000 in blocking solution for 1 h. After final washes, coverslips were treated with DAPI solution. Coverslips were fixed onto glass slides with a drop of anti-fading mounting medium and sealed with nail polish. Primary cortical cells were fixed in 10% (*v*/*v*) formalin solution (Titolchimica) for 15 min at room temperature, washed three times in PBS, and blocked in PBS containing 10% (v:v) normal goat serum (Thermo Fisher Scientific) and permeabilised with 0.3% (v:v) TritonX-100 (Merck Millipore) in PBS for 40 min. Next, cells were incubated with mouse anti-Map2 and rat anti-Gfap primary antibodies overnight at 4 °C, and after three PBS washing steps, with anti-secondary antibodies, anti-mouse IgG1 CF 568, and anti-rat Alexa Fluor 647 for 1 h at room temperature. Antibodies were diluted in PBS containing 5% (v:v) normal goat serum. Nuclei were counterstained with DAPI 1:5000 and coverslips were mounted on slides using DAKO fluorescence mounting media (Agilent, Santa Clara, CA, USA). Images at different Z-planes were collected on a Leica tcs-sp5 confocal microscope. Images were processed with the software Imaris (Bitplane AG, Belfast, UK) or ImageJ.

### 2.9. Statistical Analysis

The data are presented as the observed mean values ± SEM. The data were analysed using a 1-way ANOVA with treatment (control, insulin, glucose + insulin, insulin + insulin) as the treatment factor or 2-way ANOVA with genotype (wild-type, 3xTg-AD) and age (6, 12, and 18 months) or treatment (control, insulin, glucose + insulin, insulin + insulin). When the samples were analysed in different plates using a complete block design, an additional blocking factor plate was also included in the statistical model in order to account for any plate-to-plate variability [43]. The analyses were followed by planned comparisons of the predicted means. The analysis was performed using the InVivoStat v4.4.0 software [44]. The data were log-transformed, where appropriate, in order to stabilise the variance and satisfy the parametric assumptions. A value of *p* < 0.05 was considered statistically significant.

## 3. Results

**Figure 1:**
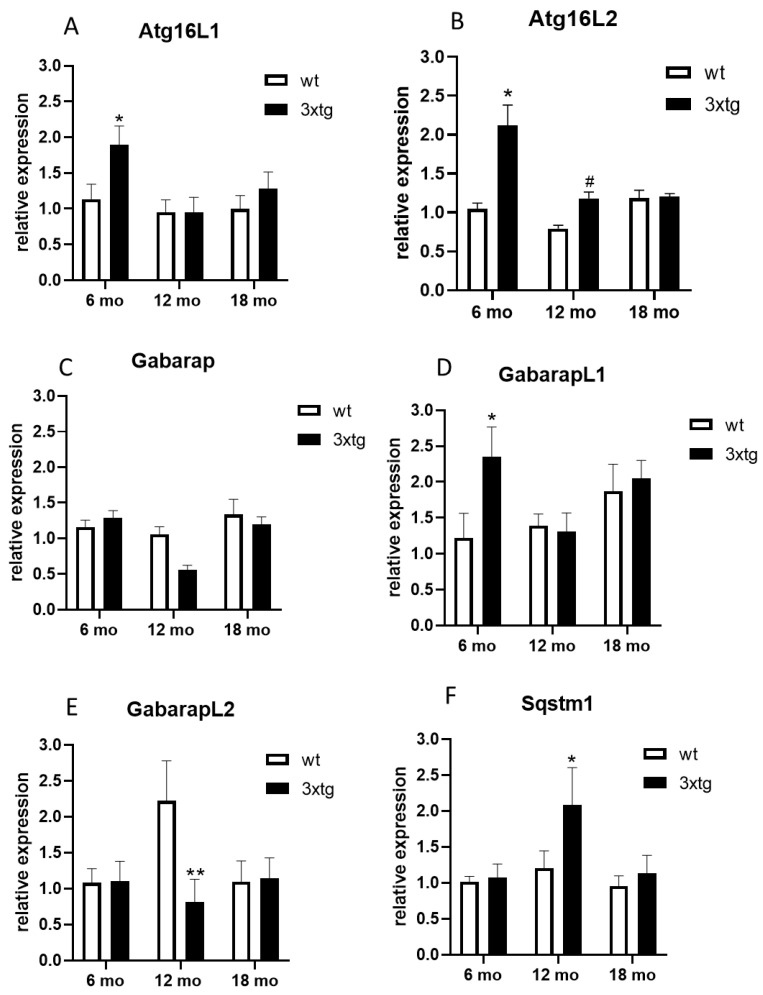
Hippocampal mRNA levels of *Atg16L1* (**A**), *Atg16L2* (**B**), *Gabarap* (**C**), *GabarapL1* (**D**), *GabarapL2* (**E**), and *Sqstm1* (**F**) in 3xTg-AD (3xTg) or wild-type (wt) mice at 6, 12, and 18 months (mo) of age. The means + SEM are plotted. **: *p* < 0.01; *: *p* < 0.05; #: 0.05 < *p* < 0.09 in the planned comparison vs. wt; n = 6/group.

Since comorbidity is frequently observed between AD and T2DM, in our previous study [26], we applied a systems biology approach to investigate if common pathophysiological alterations could be identified at a molecular level. Similar approaches had previously highlighted the role of shared cellular signalling pathways contributing to both T2DM and AD. Among them, a prominent role was discovered for neurotrophin, PI3K/AKT, MTOR, and MAPK signalling, as well as for microglial-mediated immune responses, which can cross-talk to each other [45]. In addition, our previous data revealed a central role for autophagic mechanisms; in particular, a number of autophagy-related genes were indicated as important players, namely *ATG16L1*, *ATG16L2*, *GABARAP*, *GABARAPL1*, *GABARAPL2*, and *SQSTM1*. Therefore, we first aimed to investigate whether these genes were specifically modulated in association with neurobiological alterations characterising AD. We thus analysed the expression of the respective mouse orthologues (*Atg16l1*, *Atg16l2*, *Gabarap*, *GabarapL1*, *GabarapL2*, *Sqstm1*) in a transgenic mouse model of AD. 3xTg-AD mice harbour three mutant genes for the beta-amyloid precursor protein (βAPPSwe), presenilin-1 (PS1M146V), and tauP301L [46,47]; as a consequence, the mice progressively develop plaques and tangles, as well as cognitive impairments [47,48,49]. We thus compared hippocampal gene expression between 3xTg-AD mice and the respective wild-type controls at different ages. At 6 months, *Atg16L1*, *Atg16L2*, and *GabarapL1* were expressed at significantly higher levels in 3xTg-AD mice (Figure 1A,B,D). In contrast, at 12 months, *GabarapL2* expression was significantly reduced, whereas *Sqstm1* levels were elevated (Figure 1E,F).

**Figure 2:**
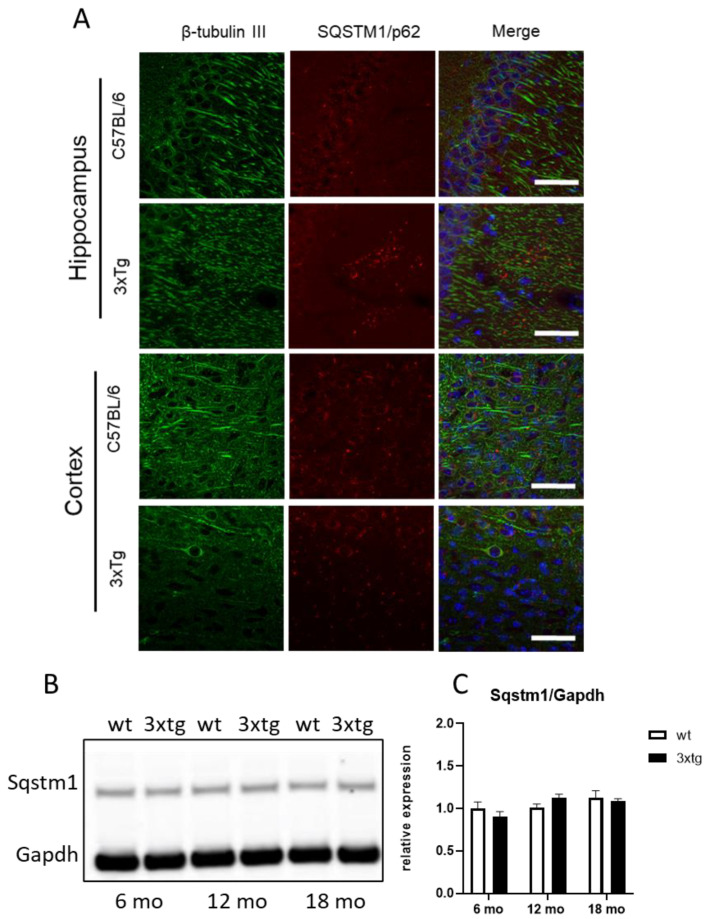
Sqstm1 and β-Tubulin III immunoreactivity in hippocampal and cortical sections of wild-type or 3xTg-AD mice at 12 months; scale bar: 20 µm (**A**). Sample Sqstm1 immunoblot (**B**). Sqstm1 levels in 3xTg-AD (3xTg) or wild-type (wt) mice at 6, 12, and 18 months (mo) of age (**C**). The means + SEM are plotted; n = 6/group.

At the protein level, although increased Sqstm1 mRNA expression was observed qualitatively in the hippocampus (Figure 2A), the increase could not be confirmed in semi-quantitative Western blotting experiments, possibly because of the lower sensitivity of the technique (Figure 2B,C).

**Figure 3:**
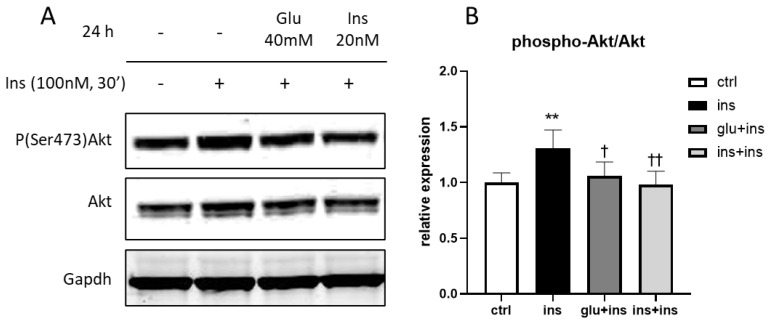
Phospho-Akt/Akt levels in response to insulin challenge (100 nM 30 min) after chronic treatment with 40 mM glucose or 20 nM insulin or vehicle. Sample immunoblot (**A**); protein levels (**B**). ** *p* < 0.01 vs. ctr; † *p* < 0.05 vs. ins; †† *p* < 0.01 vs. ins in Dunnett’s test; n = 4.

Next, we investigated whether these genes were modulated in the presence of AD-T2DM comorbidity. To model this condition, we first employed the human glioblastoma H4 cell line stably expressing the βAPP-Swe mutation [50,51,52] and applied treatments able to induce insulin resistance [53,54]. In this model, phospho-Akt/Akt levels were significantly increased by the insulin challenge (100 nM), whereas this response was abated after chronic treatment with high-concentration insulin, thus showing that insulin resistance was successfully achieved (Figure 3).

**Figure 4:**
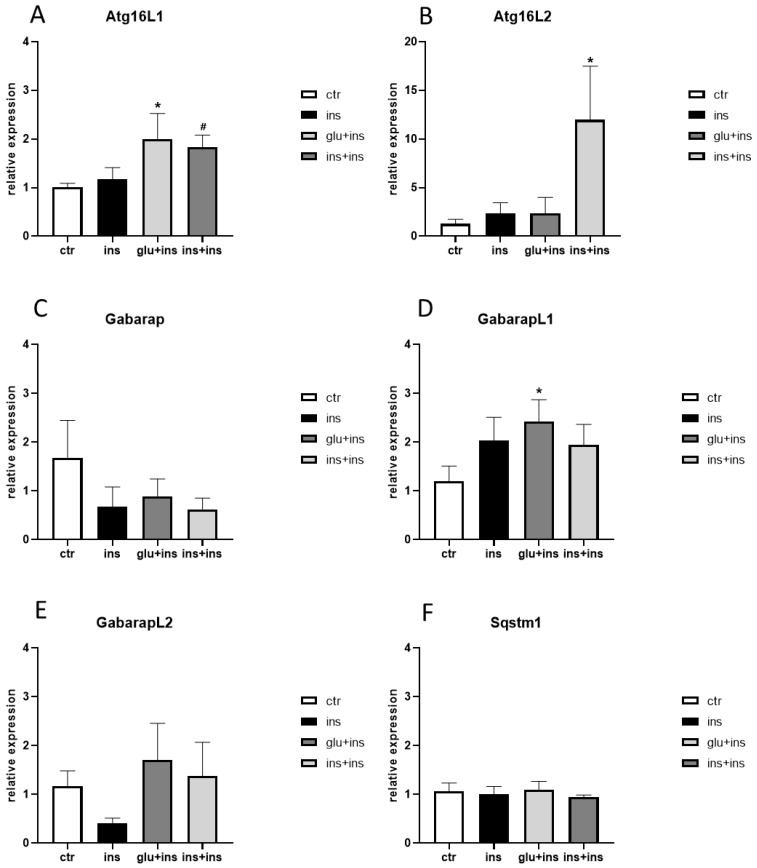
mRNA levels of *Atg16L1* (**A**), *Atg16L2* (**B**), *Gabarap* (**C**), *GabarapL1* (**D**), *GabarapL2,* (**E**) and *Sqstm1* (**F**) in H4 βAPP-Swe. Treatments were as follows: ins: 100 nM insulin for 30 min; glu + ins: 40 mM glucose for 24 h followed by 100 nM insulin for 30 min; ins + ins: 20 nM insulin for 24 h followed by 100 nM insulin for 30 min; ctr: vehicle. The means + SEM are plotted. *: *p* < 0.05; #: 0.05 < *p* < 0.09 in the planned comparison vs. ctr; n = 6/group.

Similar to findings obtained in 3xTg-AD mice, in the presence of insulin resistance, *Atg16L1*, *Atg16L2*, and *GabarapL1* expression levels were significantly increased (Figure 4A,B,D).

**Figure 5:**
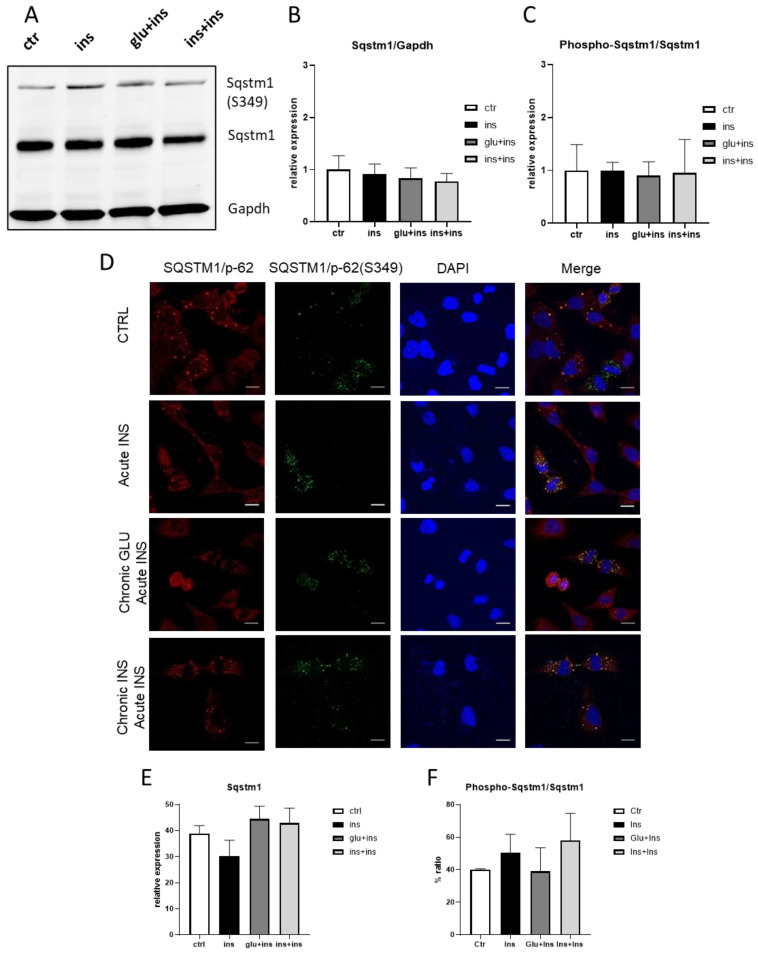
Phospho-Sqstm1 and Sqstm1 protein levels after insulin challenge (ins: 100 nM 30 min) or chronic glucose (glu + ins: 24 h 40 mM followed by 100 nM insulin 30 min) or chronic insulin (ins + ins: 24 h 20 nM insulin followed by 100 nM insulin 30 min) or vehicle (ctr) measured by Western blotting (**A**–**C**) or immunofluorescence (**D**–**F**). (**A**): sample immunoblot; (**B**): Sqstm1 levels; (**C**): phospho-Sqstm1/Sqstm1 ratio; (**D**) Sqstm1 immunofluorescence; (**E**): Sqstm1 dots/cell; (**F**): phospho-Sqstm1/Sqstm1 ratio (Scale bar: 15 µm). The means + SEM are plotted; n = 3–4.

Subsequently, we examined whether Sqstm1 phosphorylation levels were affected by the onset of insulin resistance. No significant differences were revealed by Western blot or immunofluorescence analyses (Figure 5).

**Figure 6:**
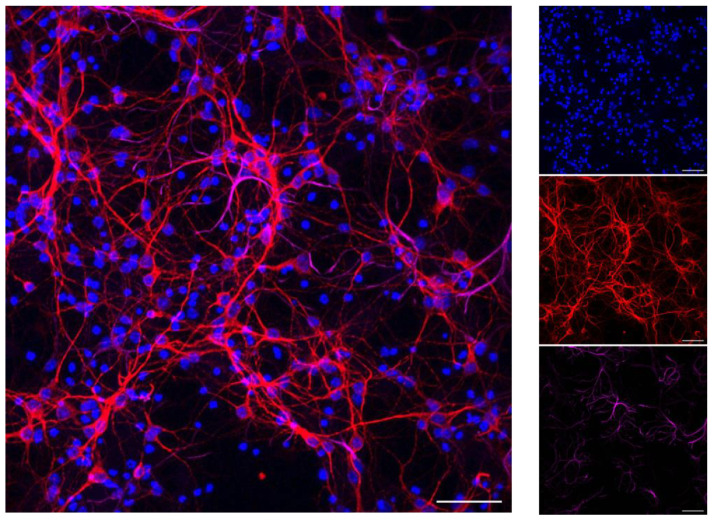
Representative images of primary mouse cortical cells maintained in culture for 7 DIV and immunolabelled for nuclear marker DAPI (blue), neuronal marker MAP2 (magenta), and astrocytic marker GFAP (magenta). The majority of cells in primary cultures were positive for MAP2 and, to a lesser degree, for GFAP. Scale bar: 50 µm.

Next, we generated a second AD-T2DM cellular model by inducing insulin resistance in neuronal primary cultures obtained from 3xTg-AD mice and wild-type controls. In this model, we confirmed that primary cultures were enriched in neurons (Figure 6).

Gene expression analysis confirmed that *Atg16L1* was significantly increased in cultures from transgenic mice when insulin resistance was induced (Table 2), whereas no other difference was detected in the other genes analysed. In addition, *Gabarap* showed a significant reduction by genotype (Table 2). However, the findings showed a very high level of variability within groups.

## 4. Discussion

In this study, we examined the modulation of genes recognised as relevant for the common cellular dysregulations sustaining the observed comorbidity between AD and T2 DM in our previous systems biology study [26]. Here, we explored their expression in 3xTg-AD mice, a transgenic mouse model of AD overexpressing mutated human genes associated with early-onset AD (*PSEN1* and *APP*) or with the formation of neurofibrillary tangles (tau) [46]. In this mouse model, the neuropathological features of AD, amyloid plaques and neurofibrillary tangles, as well as neuroinflammation, developed progressively in an age-dependent fashion. In particular, extracellular amyloid beta deposition started at six months of age and progressively increased to reach its full extent at 15 months [47,49]. Tau pathology followed a similar age-related increase, although delayed with respect to amyloid beta pathology [46,47,49]. Likewise, cognitive impairments reproducing the human pathological feature of AD appeared at six months and became progressively more severe at 12 and 20 months [49]. We discovered that at 6 months of age, *Atg16L1*, *Atg16L2*, and *GabarapL1* were expressed at higher levels in 3xTg-AD mice, whereas at later time points, this increase subsided. The alterations are in agreement with those obtained in the previous study, where pre-frontal cortex samples were analysed in two AD mice models [26]. These findings suggest that the increased expression may occur as an attempt to oppose the neuropathological alterations by activating a neuroprotective response.

A limitation of this experimental design is that 3xTg-AD mice were generated in a hybrid C57BL/6:129 genetic background; therefore, the control line we used, although similar, is not identical. However, the use of C57BL/6 as a control strain is well documented in previous studies [55,56,57,58].

To reproduce the molecular dysregulation characterising insulin resistance in AD brains, we used neuronal models of AD based either on a neuronal cell line generating amyloid beta deposits, H4Swe cells, or on the 3xTg-AD mouse primary neuron cultures. H4Swe cells are well established as tools to investigate AD-related cellular dysregulation [50,51,52]. However, a limitation is that they do not share all neuronal characteristics, being a neuroglioma-derived line. Therefore, primary neurons were also investigated. In both in vitro models, we established a condition of insulin resistance by prolonged treatment with high insulin concentrations. As a consequence, the normal response to insulin challenge is hampered by prolonged insulin exposure, and the normal Akt phosphorylation and activation responses characterising the insulin signal transduction pathway are not induced [53]. Similar to findings in 3xTg-AD mice, we found that *Atg16L1*, *Atg16L2*, and *GabarapL1* were significantly elevated in insulin resistance conditions. The increased expression of these genes in the cell model of AD-T2DM comorbidity corroborates the hypothesis of a neuroprotective role of this response, as hyperglycaemia has been previously associated with the increased beta amyloid plaque production [59].

*Atg16L1* was identified as the mammalian orthologue of the corresponding yeast gene, which was known to provide a crucial contribution to autophagic processes [60,61]. Autophagy was discovered as a process occurring in response to cellular stresses such as nutrient deprivation, infection, or hypoxia. Its chief function is providing nutrients for vital cellular activities during fasting by degrading cellular components and releasing them back to the cytoplasm to be used again. However, in addition to this non-selective approach, further studies demonstrated that autophagy can selectively eliminate potentially harmful damaged mitochondria or protein aggregates [61,62]. Consequently, autophagy dysfunction has been implicated in several diseases and its components generated interest as potential pharmacological targets [28,62]. In autophagy, starvation signals promote the recruitment of autophagy proteins to a specific subcellular location, where they assemble a structure called the phagophore. An isolation membrane is gradually formed to isolate a portion of the cytosol and is finally sealed into a vesicle, termed the autophagosome, which contains cytoplasmic material. The autophagosome then fuses with the lysosomal membrane, and the autophagic body together with its cargo are degraded [62,63]. In this process, the role of Atg16L1 is essential for autophagy initiation, as its recruitment in the Atg12-Atg5 complex is required to engage autophagic proteins in the phagophore assembly site and contribute to its scaffolding by Atg8/LC3 protein lipidation [60,64,65,66]. Therefore, the increase observed in the present study suggests an effort to trigger autophagic responses to counteract the increased production of abnormal proteins and rescue insulin response.

In addition to its well-demonstrated role in canonical autophagy, Atg16L1 was shown to exert different functions related to a structural component specifically observed in the C-terminal of the mammalian protein compared to the yeast counterpart. This specific component is necessary for the Atg16L1-mediated lipidation of single membranes, a non-canonical autophagy pathway, and specific cargo recruitment [66]. Furthermore, Atg16L1 contributes to modulating the extent of the innate immune response to injuries or infection, with an anti-inflammatory role [66,67]. Recent results showed that aged mice lacking this C-terminal domain of Atg16L1 develop beta amyloid plaques, excessive tau phosphorylation, reactive microgliosis, and memory impairments [68]. The proposed mechanism points to Atg16L1 involvement in a process defined as LANDO (LC3-associated endocytosis), which contributes to TREM2, CD36, and TLR4 recycling [68]. Therefore, the observed increased *Atg16L1* levels may contribute to establishing a protective response that goes beyond the activation of autophagic responses, but also involves a rescue from neuronal damages through different mechanisms. Interestingly, we observed increased *Atg16L1* expression in all investigated models. This result reinforces the notion of a primary role of this protein in the cellular response to both AD and T2DM pathophysiology, in a fashion independent from the in vivo or in vitro model which is well-conserved through evolution both in mice and in humans.

*Atg16L2* is a second isoform of *Atg16L1*, sharing a similar domain structure and a similar ability to bind Atg12-Atg5 and form a complex. However, the Atg16L2 protein is not recruited to phagophores and does not contribute to autophagosome formation; thus, it is not essential to canonical autophagy [69]. However, data suggesting the possibility of a cell-specific involvement in canonical autophagy are also available [70]. In addition, a recent report on the generation of *Atg16L2* knock-out mice demonstrated a contribution of this gene to the maturation of immune cells and suggested that distinct functions are associated with respect to Atg16L1 [71]. Data showing its relevance in serious diseases such as Crohn’s disease and various cancers notwithstanding, very incomplete information is available on the role of Atg16L2 [72]. Our findings also support the involvement of this widely expressed gene in the pathophysiology of insulin resistance in AD brains.

The GabarapL1 protein belongs to the Atg8/LC3 autophagy proteins, which include six members: LC3A, LC3B, LC3C, Gabarap, GabarapL1, and GabarapL2. The recruitment of Atg8 family proteins to the forming phagophore is mediated by the above-mentioned Atg12-Atg5–Atg16L1 complex and is essential for phagophore elongation and, ultimately, for autophagy [62,63,73]. GabarapL1 has also been implicated in autophagosome fusion with lysosome, and these functions are supposed to contribute to the degradation of oncogenic proteins and exert tumour-suppressive functions [73]. Interestingly, GabarapL1 has been specifically implicated in a newly discovered selective autophagy process termed glycophagy, which is involved in the transport and delivery of glycolytic fuel substrates [74]. Since these pathways regulate cellular energy demand, compelling evidence links glycophagy-mediated glucose availability to energy metabolism, in agreement with our findings.

With regard to Sqstm1 levels, contrasting findings have been previously reported. In agreement with the present results, no alterations were detected in the hippocampus or in mitochondria-enriched hippocampal fractions of young 3xTg-AD mice [75,76]. Conversely, a decrease was found in whole brain homogenates and in the mitochondria-enriched hippocampal fractions of old 3xTg-AD mice [76,77,78].

## 5. Conclusions

This study investigated the molecular underpinning of the comorbidity between AD and T2DM in cellular models of insulin resistance in the presence of AD-related neuropathological features. Our findings are in agreement with the hypothesis that impaired autophagic mechanisms are important in the pathophysiology of AD through nonstandard mechanisms. In particular, the autophagy-related genes *Atg16L1*, *Atg16L2*, and *GabarapL1* were highlighted as having a more relevant function in this mechanism, in addition to *GabarapL2* and *Sqstm1*.

## Figures and Tables

**Table 1:** Primers sequences.

Gene	Forward Primer	Reverse Primer
Mouse *Atg16L1*	GGTCTGTCCGAATCTCCCCT	GCGCATCGAAGACATACGAG
Mouse *Atg16L2*	CTGTGTGGATGTGGTGAAGG	AGCATTGACCTCAGAGAGAT
Mouse *Gabarap*	TTCTCCCCTTGTTTACCCTCCAT	TCCAATGTCAATCCCTTCCAC
Mouse *GabarapL1*	CATCGTGGAGAAGGCTCCTA	ATACAGCTGGCCCATGGTAG
Mouse *GabarapL2*	GCTATTTGCTCCAGGGAACCT	AACTATGCATCAGCCCCTCC
Mouse *Sqstm1*	GCCAGAGGAACAGATGGAGT	TCCGATTCTG GCATCTGTAG
Mouse *Gapdh*	GCCAAGGTCATCCATGACAACT	GAGGGGCCATCCACAGTCT
Mouse *Ywhaz*	TAGGTCATCGTGGAGGGTCG	GAAGCATTGGGGATCAAGAACTT
Human *ATG16L1*	ACACAAGAAACGTGGGGAGT	CTCCGTCTCCAGGTCAGAGA
Human *ATG16L2*	TTCGGGACCGTACGCAAAAG	TCAAGCTCTGACTCCTCCCA
Human *GABARAP*	CTCCCTTATTCAGGACCGGC	TGCCAACTCCACCATTACCC
Human *GABARAPL1*	TACCTAGTGCCCTCTGACCT	TGGACGGCATCCAGTATTGT
Human *GABARAPL2*	GCGAAATATCCCGACAGGGT	TCCACATGAACTGAGCCACA
Human *SQSTM1*	GAGATTCGCCGCTTCAGCTT	GGAAAAGGCAACCAAGTCCC
Human *GAPDH*	CCATGGGGAAGGTGAAGGTC	TGGAATTTGCCATGGGTGGA
Human *YWHAZ*	GGACTACGACGTCCCTCAAA	CCAGTTTGGCCTTCTGAACC

**Table 2:** mRNA levels of *Atg16L1*, *Atg16L2*, *Gabarap*, *GabarapL1*, *GabarapL2*, and *Sqstm1* in neuronal primary cultures obtained from wild-type or 3xTg-AD mice. Cells received insulin treatment (ins) at 100 nM for 30 min; glucose at 40 mM for 24 h followed by 100 nM insulin for 30 min (glu + ins); insulin at 20 nM for 24 h followed by 100 nM insulin 30 min (ins + ins); or vehicle (ctr). The means ± SEM are reported. *: *p* < 0.05 in the planned comparison vs. ctr; n = 3/group.

	Wild-Type				3xTg-AD			
	ctr	ins	glu + ins	ins + ins	ctr	ins	glu + ins	ins + ins
*Atg16L1*	1.51 ± 0.76	2.18 ± 1.25	0.15 ± 0.04	0.16 ± 0.06	0.18 ± 0.11	0.95 ± 0.92	3.03 ± 1.99 *	0.77 ± 0.47
*Atg16L2*	1.14 ± 0.34	0.41 ± 0.07	1.14 ± 0.34	1.04 ± 0.54	0.88 ± 0.69	0.92 ± 0.68	1.07 ± 0.54	0.44 ± 0.27
*Gabarap*	1.13 ± 0.27	1.30 ± 0.47	0.95 ± 0.17	0.76 ± 0.13	0.64 ± 0.14	0.46 ± 0.15	0.63 ± 0.23	0.46 ± 0.21
*GabarapL1*	1.05 ± 0.23	0.91 ± 0.23	0.87 ± 0.07	0.86 ± 0.30	1.31 ± 0.54	1.02 ± 0.45	0.81 ± 0.32	1.30 ± 0.25
*GabarapL2*	1.17 ± 0.31	1.56 ± 0.38	1.03 ± 0.19	1.49 ± 0.39	1.32 ± 0.56	0.63 ± 0.29	1.51 ± 0.69	0.88 ± 0.47
*Sqstm1*	1.00 ± 0.05	0.74 ± 0.11	0.99 ± 0.15	0.66 ± 0.19	0.74 ± 0.32	0.79 ± 0.39	0.83 ± 0.35	0.60 ± 0.23

## Data Availability

Data are shown in the manuscript. Additional details can be requested from the authors.
